# Supplementary material for: Transplacental Innate Immune Training via Maternal Microbial Exposure: Role of XBP1-ERN1 Axis in Dendritic Cell Precursor Programming
Source: Front Immunol. 2020 Dec 2;11:601494. doi: 10.3389/fimmu.2020.601494 (PMC7793790; doi:10.3389/fimmu.2020.601494)
Supplement: Supplementary file 1 [file DataSheet_1.pdf]

Supplementary Material

Supplementary Table 1. Differentially expressed genes comparing bone marrow of fetuses from OM-85 treated versus untreated mothers.

GeneID	Symbol	baseMean	log2FC	lfcSE	stat	p-value	adjusted p-value
231070	Insig1	1227.87	0.31	0.08	3.76	1.73E-04	5.99E-02
319554	Idi1	297.74	0.29	0.07	3.99	6.63E-05	4.00E-02
78894	Aacs	1184.93	0.28	0.07	4.23	2.32E-05	2.66E-02
64136	Sdf2l1	470.14	0.28	0.08	3.58	3.38E-04	7.01E-02
240025	Dact2	1114.16	0.28	0.08	3.35	7.97E-04	9.05E-02
16835	Ldlr	2953.52	0.27	0.07	3.67	2.47E-04	6.78E-02
71911	Bdh1	914.76	0.27	0.08	3.34	8.45E-04	9.05E-02
19273	Ptpru	284.90	0.25	0.08	3.25	1.14E-03	9.46E-02
331026	Gmppb	548.39	0.24	0.06	3.95	7.92E-05	4.11E-02
56325	Abcb9	518.14	0.24	0.07	3.56	3.77E-04	7.01E-02
76737	Creld2	834.95	0.24	0.05	4.72	2.40E-06	1.37E-02
74246	Gale	1123.69	0.23	0.06	4.20	2.67E-05	2.66E-02
71609	Tradd	312.97	0.23	0.06	4.16	3.24E-05	2.66E-02
192156	Mvd	666.21	0.23	0.06	3.71	2.10E-04	6.48E-02
59031	Chst12	813.08	0.22	0.07	3.38	7.32E-04	9.00E-02
74840	Manf	2133.29	0.22	0.05	4.35	1.36E-05	2.32E-02
17428	Mnt	1032.31	0.21	0.06	3.49	4.80E-04	7.50E-02

GeneID	Symbol	baseMean	log2FC	lfcSE	stat	p-value	adjusted p-value
233912	Armc5	603.81	0.20	0.06	3.61	3.03E-04	7.01E-02
13360	Dhcr7	1513.17	0.20	0.06	3.33	8.54E-04	9.05E-02
15015	H2-Q4	790.97	0.19	0.06	3.27	1.08E-03	9.24E-02
20787	Srebf1	4157.10	0.19	0.05	3.49	4.87E-04	7.50E-02
68671	Pcyt2	1658.43	0.19	0.04	4.81	1.48E-06	1.37E-02
72296	Rusc1	541.78	0.19	0.05	3.84	1.21E-04	5.11E-02
56470	Rgs19	955.91	0.18	0.04	4.38	1.21E-05	2.32E-02
230145	Galnt12	574.23	0.18	0.05	3.92	8.80E-05	4.37E-02
56473	Fads2	1749.89	0.18	0.05	3.79	1.51E-04	5.76E-02
235293	Sc5d	1018.49	0.18	0.05	3.76	1.67E-04	5.96E-02
320534	Tmem104	400.07	0.18	0.05	3.40	6.72E-04	8.62E-02
13358	Slc25a1	769.68	0.18	0.05	3.23	1.23E-03	9.83E-02
12317	Calr	9484.29	0.18	0.05	3.65	2.62E-04	6.78E-02
67065	Polr3d	801.45	0.17	0.05	3.67	2.40E-04	6.78E-02
225849	Ppp2r5b	654.98	0.17	0.05	3.56	3.73E-04	7.01E-02
12915	Atf6b	1644.33	0.17	0.05	3.35	8.16E-04	9.05E-02
228368	Slc35c1	1022.13	0.17	0.04	4.20	2.70E-05	2.66E-02
18799	Plcd1	1381.75	0.17	0.05	3.36	7.73E-04	9.00E-02
103724	Tbc1d10a	827.76	0.17	0.05	3.43	5.99E-04	8.00E-02

GeneID	Symbol	baseMean	log2FC	lfcSE	stat	p-value	adjusted p-value
109815	Vimp	368.39	0.16	0.05	3.25	1.15E-03	9.46E-02
57377	Mogs	2743.90	0.16	0.04	3.65	2.62E-04	6.78E-02
64144	Mllt1	2427.05	0.16	0.05	3.30	9.83E-04	9.16E-02
26394	Lypla2	1464.47	0.16	0.05	3.46	5.36E-04	7.80E-02
72056	1810055G02Rik	1035.38	0.16	0.04	3.72	2.02E-04	6.46E-02
72029	Cnpy3	1174.74	0.16	0.04	4.25	2.16E-05	2.66E-02
103425	Ncln	2243.69	0.16	0.05	3.31	9.35E-04	9.14E-02
269881	Map3k10	498.33	0.16	0.05	3.38	7.25E-04	9.00E-02
74126	Syvn1	1907.98	0.16	0.05	3.22	1.29E-03	9.99E-02
23991	Cib1	731.86	0.15	0.04	3.65	2.67E-04	6.78E-02
73062	Ppp1r16a	739.65	0.15	0.04	3.87	1.09E-04	4.80E-02
68017	Mrm2	213.39	0.15	0.05	3.28	1.04E-03	9.18E-02
93685	Entpd7	521.74	0.15	0.04	3.33	8.72E-04	9.05E-02
107242	AI837181	1237.54	0.15	0.04	3.54	3.93E-04	7.01E-02
23917	Impdh1	2864.39	0.15	0.04	3.36	7.69E-04	9.00E-02
12304	Pdia4	4125.69	0.14	0.04	3.28	1.03E-03	9.18E-02
270066	Slc35e1	2671.84	0.14	0.03	4.42	9.91E-06	2.32E-02
277010	Marveld1	2058.69	0.14	0.04	3.21	1.33E-03	9.99E-02
70314	Rabep2	533.66	0.14	0.04	3.52	4.32E-04	7.01E-02
67789	Dalrd3	865.66	0.14	0.04	3.21	1.31E-03	9.99E-02

GeneID	Symbol	baseMean	log2FC	lfcSE	stat	p-value	adjusted p-value
234378	Klhl26	658.37	0.14	0.04	3.28	1.03E-03	9.18E-02
223690	Ankrd54	788.15	0.14	0.04	3.55	3.81E-04	7.01E-02
231807	BC037034	1012.51	0.14	0.04	3.63	2.89E-04	7.01E-02
664994	Isoc2a	457.74	0.14	0.04	3.65	2.60E-04	6.78E-02
71853	Pdia6	7221.20	0.14	0.04	3.59	3.26E-04	7.01E-02
18081	Ninj1	439.39	0.14	0.04	3.21	1.32E-03	9.99E-02
208638	Slc25a38	505.11	0.14	0.04	3.29	9.98E-04	9.16E-02
22433	Xbp1	3609.25	0.14	0.04	3.58	3.47E-04	7.01E-02
26433	Plod3	2737.30	0.14	0.04	3.23	1.22E-03	9.83E-02
56214	Scamp4	1189.97	0.14	0.04	3.60	3.22E-04	7.01E-02
66058	Tmem176a	1019.93	0.14	0.04	3.29	9.94E-04	9.16E-02
20818	Srprb	1450.32	0.14	0.04	3.54	3.98E-04	7.01E-02
503610	Zdhhc18	1356.49	0.14	0.04	3.57	3.61E-04	7.01E-02
70231	Gorasp2	3387.38	0.13	0.04	3.46	5.40E-04	7.80E-02
106200	Txndc11	934.54	0.13	0.03	3.99	6.65E-05	4.00E-02
74451	Pgs1	876.13	0.13	0.03	3.89	1.00E-04	4.73E-02
68427	Slc39a13	1519.83	0.13	0.04	3.37	7.47E-04	9.00E-02
217664	Mgat2	1853.11	0.13	0.04	3.45	5.58E-04	7.85E-02
22232	Slc35a2	1120.66	0.13	0.04	3.52	4.36E-04	7.01E-02

GeneID	Symbol	baseMean	log2FC	lfcSE	stat	p-value	adjusted p-value
22687	Zpr1	1098.39	0.13	0.03	4.03	5.50E-05	3.69E-02
100090	Zbtb48	459.73	0.13	0.04	3.26	1.11E-03	9.42E-02
76267	Fads1	3584.00	0.13	0.03	4.34	1.42E-05	2.32E-02
71116	Stx18	603.81	0.13	0.03	4.14	3.50E-05	2.66E-02
218271	B4galt7	571.18	0.13	0.04	3.29	1.00E-03	9.16E-02
53421	Sec61a1	6887.06	0.12	0.03	3.77	1.66E-04	5.96E-02
68385	Tlcd1	319.70	0.12	0.04	3.31	9.38E-04	9.14E-02
20405	Sh3gl1	2885.28	0.12	0.04	3.43	6.02E-04	8.00E-02
54399	Bet1l	782.18	0.12	0.03	3.53	4.11E-04	7.01E-02
76025	Cant1	1650.55	0.12	0.03	3.96	7.36E-05	4.03E-02
20514	Slc1a5	1765.32	0.12	0.03	3.43	6.08E-04	8.00E-02
11993	Aup1	1989.09	0.12	0.03	3.47	5.29E-04	7.80E-02
28106	Mydgf	1263.52	0.12	0.03	3.37	7.63E-04	9.00E-02
20498	Slc12a4	2174.73	0.12	0.04	3.31	9.44E-04	9.14E-02
20832	Ssr4	1760.63	0.12	0.03	3.53	4.12E-04	7.01E-02
19246	Ptpn1	1994.99	0.12	0.03	3.45	5.64E-04	7.85E-02
66357	Ostc	2242.03	0.12	0.03	3.57	3.62E-04	7.01E-02
72727	B3gat3	1046.49	0.11	0.04	3.22	1.26E-03	9.94E-02
56530	Cnpy2	1775.08	0.11	0.03	3.48	4.93E-04	7.50E-02
66059	Krtcap2	1263.12	0.11	0.03	3.37	7.43E-04	9.00E-02

GeneID	Symbol	baseMean	log2FC	lfcSE	stat	p-value	adjusted p-value
74504	Fam53a	728.64	0.11	0.03	3.26	1.11E-03	9.42E-02
73836	Slc35b2	1774.75	0.11	0.03	3.53	4.23E-04	7.01E-02
52858	Cdipt	1544.68	0.11	0.03	4.14	3.43E-05	2.66E-02
17308	Mgat1	2619.94	0.11	0.03	3.62	2.96E-04	7.01E-02
66156	Anapc11	977.77	0.11	0.03	3.31	9.30E-04	9.14E-02
56457	Clptm1	2346.11	0.11	0.03	3.44	5.89E-04	8.00E-02
68047	Mpnd	1339.91	0.11	0.03	3.42	6.21E-04	8.06E-02
71667	Tmem248	1805.98	0.10	0.03	3.45	5.58E-04	7.85E-02
13852	Stx2	934.84	0.10	0.03	3.53	4.16E-04	7.01E-02
20932	Surf4	8479.03	0.10	0.03	3.71	2.04E-04	6.46E-02
68090	Yif1a	900.31	0.10	0.03	3.55	3.79E-04	7.01E-02
14792	Lpcat3	1414.89	0.10	0.03	4.07	4.72E-05	3.37E-02
76479	Smndc1	1143.61	0.10	0.03	3.33	8.60E-04	9.05E-02
84095	Pi4k2a	1255.74	0.10	0.03	3.27	1.07E-03	9.24E-02
67511	Tmed9	2708.52	0.10	0.03	3.83	1.28E-04	5.23E-02
11867	Arpc1b	2894.10	0.10	0.03	3.48	5.07E-04	7.62E-02
236732	Rbm10	1178.82	0.10	0.03	3.24	1.18E-03	9.58E-02
20333	Sec22b	2051.88	0.10	0.03	3.43	6.09E-04	8.00E-02
12313	Calm1	10795.59	0.09	0.03	3.33	8.68E-04	9.05E-02

GeneID	Symbol	baseMean	log2FC	lfcSE	stat	p-value	adjusted p-value
72055	Slc38a10	4184.42	0.09	0.03	3.37	7.60E-04	9.00E-02
64143	Ralb	880.29	0.09	0.03	3.23	1.25E-03	9.90E-02
68944	Tmco1	877.50	0.09	0.03	3.52	4.32E-04	7.01E-02
11848	Rhoa	4109.43	0.08	0.02	3.32	8.92E-04	9.14E-02
20529	Slc31a1	1843.88	0.07	0.02	3.33	8.64E-04	9.05E-02
75717	Cul5	1551.18	-0.08	0.02	-3.49	4.77E-04	7.50E-02
19317	Qk	6579.70	-0.09	0.03	-3.33	8.60E-04	9.05E-02
380916	Lrch1	1181.85	-0.10	0.03	-3.70	2.18E-04	6.54E-02
59125	Nek7	1619.69	-0.11	0.03	-3.73	1.91E-04	6.40E-02
81003	Trim23	702.07	-0.12	0.03	-3.60	3.17E-04	7.01E-02
381306	BC055324	684.71	-0.13	0.04	-3.22	1.28E-03	9.99E-02
14594	Ggta1	2022.88	-0.13	0.04	-3.34	8.33E-04	9.05E-02
103573	Xpo1	8600.19	-0.13	0.04	-3.25	1.14E-03	9.46E-02
20843	Stag2	6488.16	-0.14	0.04	-3.32	9.03E-04	9.14E-02
433931	Pigg	382.49	-0.14	0.04	-3.28	1.05E-03	9.18E-02
245631	Mum1l1	1048.22	-0.15	0.04	-3.34	8.26E-04	9.05E-02
26399	Map2k6	453.63	-0.15	0.04	-3.34	8.37E-04	9.05E-02
211329	Ncoa7	568.38	-0.15	0.04	-3.69	2.24E-04	6.56E-02
329260	Dennd1b	874.05	-0.15	0.05	-3.32	9.14E-04	9.14E-02
76967	2700049A03Rik	775.57	-0.15	0.05	-3.25	1.15E-03	9.46E-02

GeneID	Symbol	baseMean	log2FC	lfcSE	stat	p-value	adjusted p-value
18583	Pde7a	1257.44	-0.15	0.04	-3.81	1.37E-04	5.38E-02
78796	Zcchc4	234.07	-0.16	0.05	-3.28	1.04E-03	9.18E-02
17957	Napb	219.35	-0.16	0.05	-3.21	1.30E-03	9.99E-02
230259	E130308A19Rik	261.16	-0.16	0.05	-3.39	7.11E-04	9.00E-02
229473	D930015E06Rik	5508.16	-0.17	0.04	-4.15	3.28E-05	2.66E-02
54598	Calcrl	1124.86	-0.18	0.06	-3.29	9.87E-04	9.16E-02
14007	Celf2	5372.06	-0.19	0.06	-3.29	1.00E-03	9.16E-02
54610	Tbc1d8	732.43	-0.20	0.05	-3.56	3.76E-04	7.01E-02
78286	Nav2	3091.40	-0.21	0.06	-3.54	3.98E-04	7.01E-02
12894	Cpt1a	2270.83	-0.23	0.06	-3.96	7.42E-05	4.03E-02
14051	Eya4	664.37	-0.24	0.07	-3.27	1.08E-03	9.24E-02
241589	D430041D05Rik	864.80	-0.26	0.08	-3.21	1.32E-03	9.99E-02
77963	Hook1	626.22	-0.27	0.08	-3.24	1.18E-03	9.58E-02
72685	Dnajc6	961.70	-0.28	0.08	-3.31	9.41E-04	9.14E-02
17472	Gbp4	1119.76	-0.32	0.10	-3.30	9.53E-04	9.14E-02
12823	Col19a1	243.16	-0.33	0.09	-3.88	1.04E-04	4.73E-02
57875	Angptl4	338.30	-0.35	0.10	-3.59	3.34E-04	7.01E-02
11535	Adm	179.61	-0.51	0.11	-4.53	5.90E-06	2.25E-02

Supplementary Table 2. Top 15 InnateDB pathways of differentially expressed upregulated genes comparing bone marrow of fetuses from OM-85 treated versus untreated mothers.

Pathway Name (no. of genes)	Gene Symbols	Adjusted *P*-value
**Protein processing in endoplasmic reticulum (n=10)**	*Atf6b, Calr, Mogs, Pdia4, Pdia6, Sec61a1, Ssr4, Syvn1, Vimp, Xbp1*	1.67E-05
**Transport of nucleotide sugars (n=3)**	*Slc35a2, Slc35b2, Slc35c1*	3.52E-04
**Cholesterol biosynthesis (n=4)**	*Dhcr7, Idi1, Mvd, Sc5d*	4.27E-04
**Regulation of cholesterol biosynthesis by SREBP (SREBF) (n=5)**	*Dhcr7, Idi1, Insig1, Mvd, Sc5d*	4.55E-04
**Metabolism of lipids and lipoproteins (n=14)**	*Bdh1, Cdipt, Dhcr7, Fads1, Fads2, Idi1, Insig1, Ldlr, Lpcat3, Mvd, Pcyt2, Pgs1, Pi4k2a, Sc5d*	4.59E-04
**Unfolded Protein Response (UPR) (n=5)**	*Calr, D17Wsu104e, Syvn1, Xbp1, Yif1a*	8.65E-04
**SNARE interactions in vesicular transport (n=4)**	*Bet1l, Sec22b, Stx18, Stx2*	9.36E-04
**Activation of gene expression by SREBF (SREBP) (n=4)**	*Dhcr7, Idi1, Mvd, Sc5d*	9.48E-04
**Asparagine N-linked glycosylation (n=6)**	*Calr, Gmppb, Mgat1, Mgat2, Mogs, Mvd*	9.57E-04
**Metabolism of proteins (n=13)**	*Calr, D17Wsu104e, Galnt12, Gmppb, Mgat1, Mgat2, Mogs, Mvd, Sec61a1, Ssr4, Syvn1, Xbp1, Yif1a*	9.86E-04
**XBP1(S) activates chaperone genes (n=4)**	*D17Wsu104e, Syvn1, Xbp1, Yif1a*	1.61E-03
**IRE1alpha activates chaperones (n=4)**	*D17Wsu104e, Syvn1, Xbp1, Yif1a*	1.62E-03
**Metabolism (n=21)**	*B3gat3, B4galt7, Bdh1, Cdipt, Chst12, Dhcr7, Fads1, Fads2, Gale, Idi1, Impdh1, Insig1, Ldlr, Lpcat3, Mvd, Pcyt2, Pgs1, Pi4k2a, Plcd1, Sc5d, Slc35b2*	2.31E-03
**Glycosaminoglycan biosynthesis (n=3)**	*B3gat3, B4galt7, Chst12*	2.41E-03
**Post-translational protein modification (n=7)**	*Calr, Galnt12, Gmppb, Mgat1, Mgat2, Mogs, Mvd*	2.50E-03

Supplementary Table 3. Top 20 upstream regulators of differentially expressed genes comparing bone marrow of fetuses from OM-85 treated versus untreated mothers.

Upstream Regulator	Molecule Type	Predicted Activation State	Target Genes	Activation z-score	*P*-value
**XBP1**	Transcription regulator	Activated	*ATF6B, BET1L, CALR, GORASP2, MGAT2, MOGS, PDIA4, PDIA6, SDF2L1, SEC22B, SEC61A1, SREBF1, SRPRB, SSR4, STX18, SURF4, SYVN1, TXNDC11, XBP1, YIF1A*	4.427	3.81E-17
**SCAP**	Other	Activated	*AACS, DHCR7, FADS2, IDI1, INSIG1, LDLR, MVD, SC5D, SREBF1*	2.949	1.52E-10
**SREBF2**	Transcription regulator	Activated	*AACS, DHCR7, FADS2, IDI1, INSIG1, LDLR, MVD, SC5D, SREBF1*	2.745	2.95E-09
**INSIG1**	Other	Inhibited	*AACS, DHCR7, FADS1, FADS2, IDI1, LDLR, LPCAT3, PCYT2, SREBF1*	-2.931	5.81E-08
**POR**	Enzyme	Inhibited	*BDH1, CPT1A, DHCR7, FADS2, HLA-A, IDI1, INSIG1, LDLR, MVD, SC5D, TMEM176A*	-2.8	8.67E-08
**SIRT2**	Transcription regulator	Activated	*AACS, DHCR7, IDI1, MVD, SC5D*	2.236	1.52E-07
**SREBF1**	Transcription regulator	Activated	*AACS, DHCR7, FADS1, FADS2, IDI1, INSIG1, LDLR, MVD, SC5D, SREBF1*	3.056	1.95E-06
**ERN1**	Kinase	Activated	*HOOK1, MVD, SDF2L1, SEC22B, SEC61A1, SURF4, SYVN1, XBP1*	2.156	3.32E-06
**CD38**	Enzyme	Activated	*B4GALT7, CHST12, CRELD2, MANF, PDIA6, SDF2L1, XBP1*	2.588	1.48E-04
**NFE2L2**	Transcription regulator	Activated	*Calm1, DHCR7, IMPDH1, MOGS, PDIA4, PDIA6, PTPN1, SEC61A1, Slc35a2, SREBF1, XBP1*	2.111	1.68E-04
**miR-874-5p**	Mature microrna	Inhibited	*ANGPTL4, FAM53A, GALE, KLHL26, LPCAT3, MARVELD1, MNT, NCLN, SCAMP4, SLC35C1, SLC39A13, TMEM104*	-2.887	1.88E-04
**miR-4731-5p**	Mature microrna	Inhibited	*ARMC5, BET1L, CALR, DACT2, LYPLA2, MNT, NAV2, NINJ1, QKI,*	-2.84	3.83E-04

			SCAMP4, SH3GL1, SLC35E1, SLC39A13, SYVN1, ZDHHC18		
**SLC13A1**	Transporter	**Inhibited**	*GALE, INSIG1, MVD, SDF2L1, XBP1*	-2.236	5.65E-04
**IL5**	Cytokine	**Activated**	*CHST12, CRELD2, IDI1, MANF, PDIA6, SDF2L1, SLC1A5, XBP1*	2.758	1.07E-03
**miR-4503**	Mature microrna	**Inhibited**	*C11orf24, IDI1, SC5D, SYVN1*	-2	1.62E-03
**miR-6887-3p**	Mature microrna	**Inhibited**	*ARPC1B, BDH1, CNPY2, EYA4, FADS2, IMPDH1, ISOC2, MNT, MOGS, PDE7A, SURF4, TMED9*	-2.309	1.72E-03
**SIRT1**	Transcription regulator	**Inhibited**	*CPT1A, LDLR, MGAT1, MGAT2, MLLT1, SEC61A1, SREBF1*	-2.395	1.96E-03
**miR-6132**	Mature microrna	**Inhibited**	*ARMC5, IMPDH1, MAP3K10, MNT, NINJ1, SH3GL1, SLC25A38, SLC35B2, SLC39A13, TMCO1, TMED9, TMEM176A*	-3.464	2.86E-03
**miR-149-3p**	Mature microrna	**Inhibited**	*ANKRD54, BET1L, C7orf43, CALR, CNPY3, FADS2, KLHL26, MARVELD1, MNT, NAV2, RABEP2, RALB, RUSC1, SCAMP4, SH3GL1, SLC35B2, STX2, TMEM104*	-3.771	2.99E-03
**miR-6795-5p**	Mature microrna	**Inhibited**	*BET1L, C11orf68, FADS2, ISOC2, KLHL26, LYPLA2, MNT, MOGS, PI4K2A, POLR3D, RGS19, SCAMP4, SYVN1*	-3.606	3.03E-03

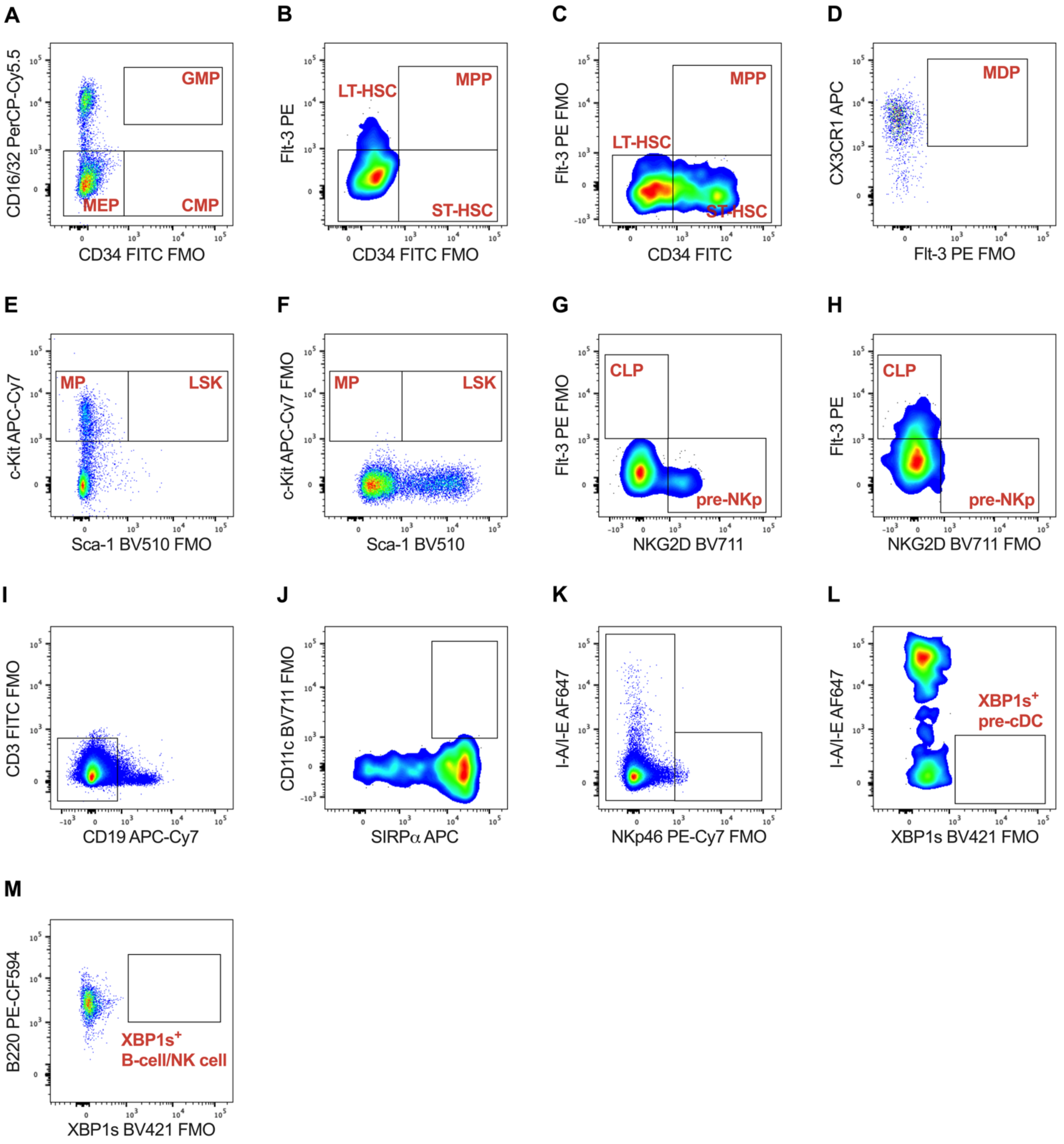

Supplementary Figure 1. Fluorescence minus one (FMO) staining controls. Terminal population gates and intermediate gates where required for the **(A-H)** HSPC staining panel, **(I-J)** dendritic cell staining panel and **(K-M)** XBP1s staining panel. GMP = granulocyte-macrophage progenitor; MEP = megakaryocyte erythrocyte progenitor; CMP = common myeloid progenitor; LT-HSC = long-term hematopoietic stem cell; ST-HSC = short-term hematopoietic stem cell; MPP = multipotent progenitor; MDP = macrophage-dendritic cell progenitor; MP = myeloid progenitor; LSK = Lin$^-$c-Kit$^-$Sca-1$^-$; CLP = common lymphoid progenitor; pre-NKp = pre-natural killer cell progenitor.

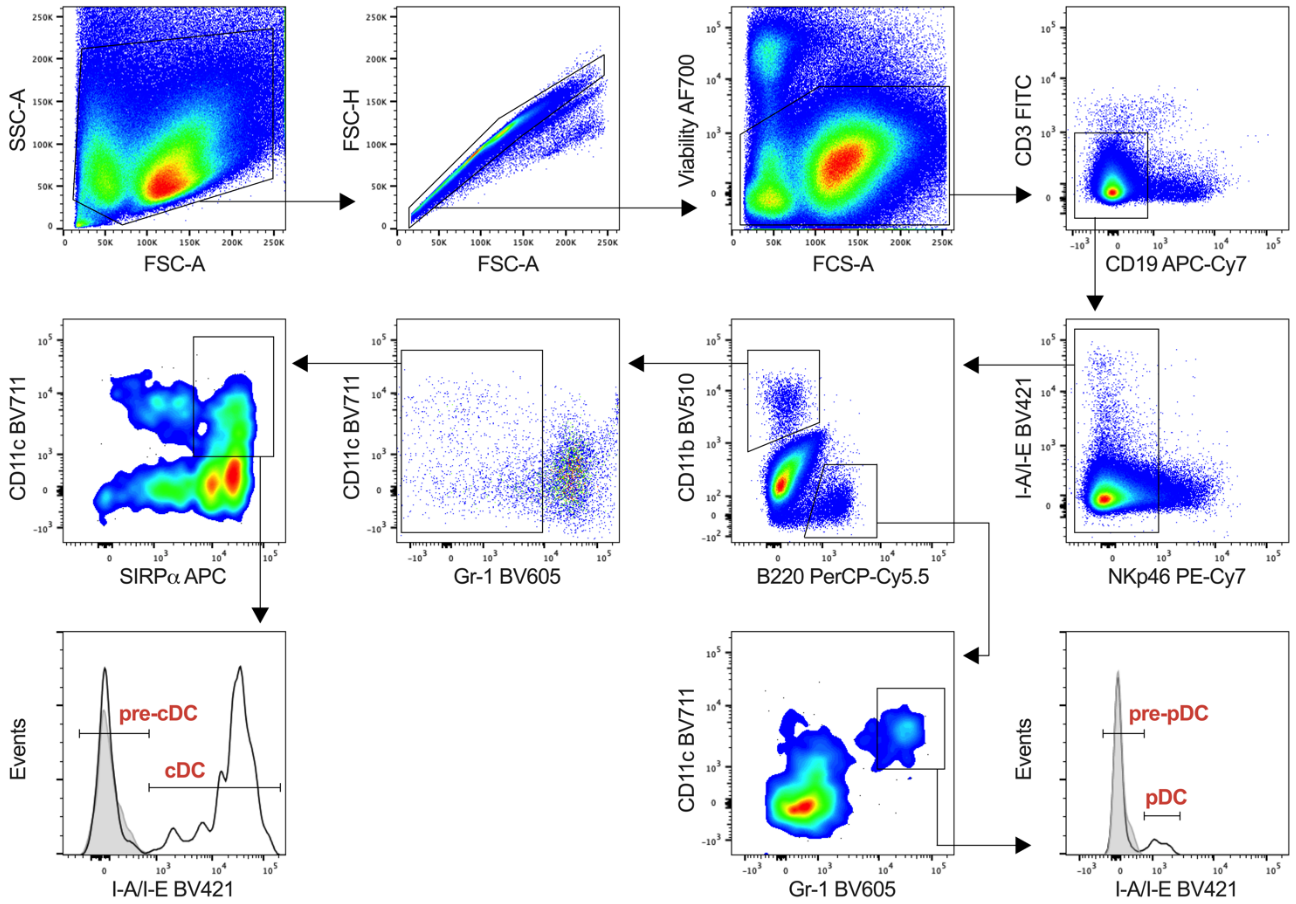

Supplementary Figure 2. Fetal bone marrow dendritic cell gating strategy. Gating strategy used to immunophenotypically characterize dendritic cell populations within fBM. Shaded area of the histogram represents I-A/I-E BV421 FMO control.

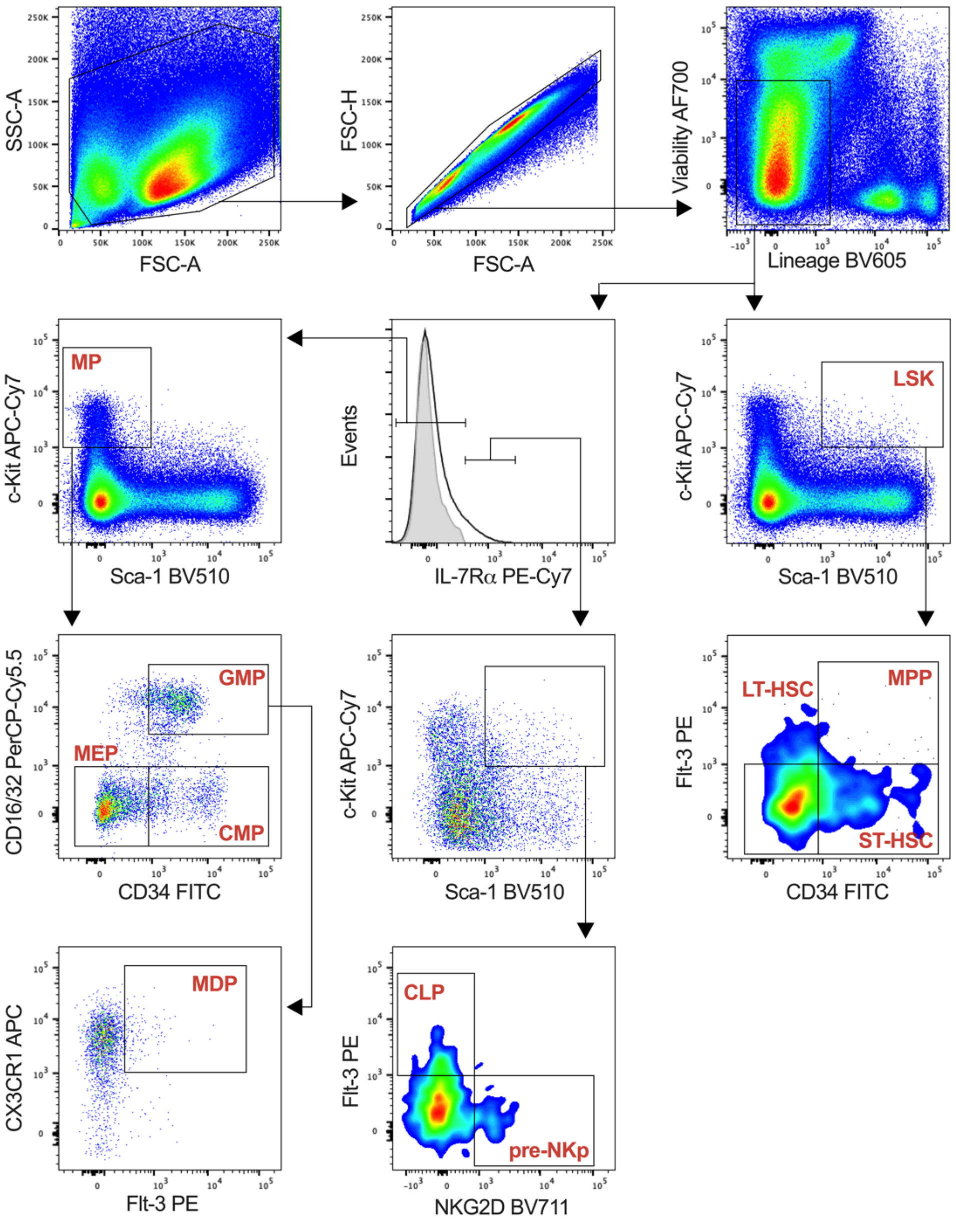

Supplementary Figure 3. Fetal bone marrow hematopoietic stem and progenitor cell gating strategy. Gating strategy used to immunophenotypically characterize HSPC populations within fBM. Lineage gate = $CD2^{-}CD3^{-}CD4^{-}CD5^{-}CD8\alpha^{-}CD19^{-}B220^{-}Gr\text{-}1^{-}Ter119^{-}$. Shaded area of the histogram represents IL-7Rα PE-Cy7 FMO control. MP = myeloid progenitor; LSK = $Lin^{-}c\text{-}Kit^{-}Sca\text{-}1^{-}$; GMP = granulocyte-macrophage progenitor; MEP = megakaryocyte erythrocyte progenitor; CMP = common myeloid progenitor; MDP = macrophage-dendritic cell progenitor; LT-HSC = long-term hematopoietic stem cell; ST-HSC = short-term hematopoietic stem cell; MPP = multipotent progenitor; CLP = common lymphoid progenitor; pre-NKp = pre-natural killer cell progenitor.

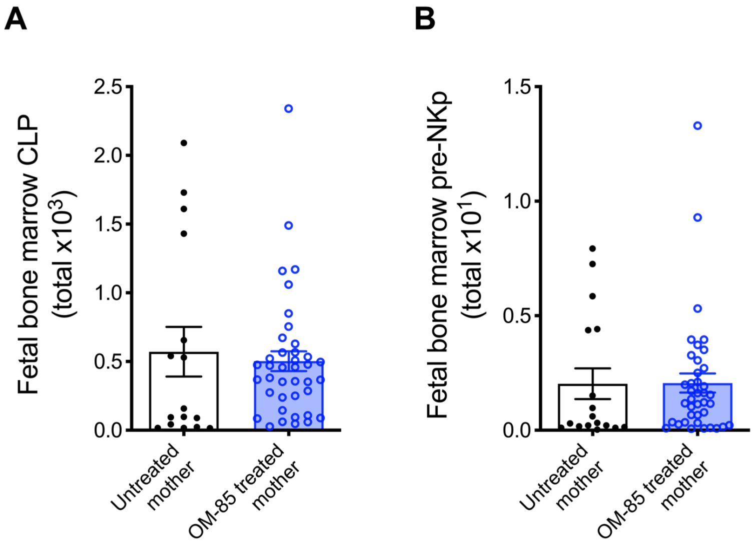

Supplementary Figure 4. Total CLP and pre-NKp within fetal bone marrow following maternal OM-85 treatment. Absolute numbers of **(A)** $Lin^{-}IL\text{-}7R\alpha^{+}c\text{-}Kit^{+}Sca\text{-}1^{+}Flt\text{-}3^{+}$ common lymphoid progenitors (CLP) and **(B)** $Lin^{-}IL\text{-}7R\alpha^{+}c\text{-}Kit^{+}Sca\text{-}1^{+}Flt\text{-}3^{-}NKG2D^{+}$ pre-natural killer cell progenitors (pre-NKp) in fetal bone marrow. Data are presented from individual animals comparing fetuses from OM-85-treated and untreated mothers and displayed as bar graphs showing mean ± SEM of n = 8 independent experiments. Statistical significance was assessed by Mann-Whitney U test based on distribution of the data as determined by D'Agostino-Pearson omnibus normality test.

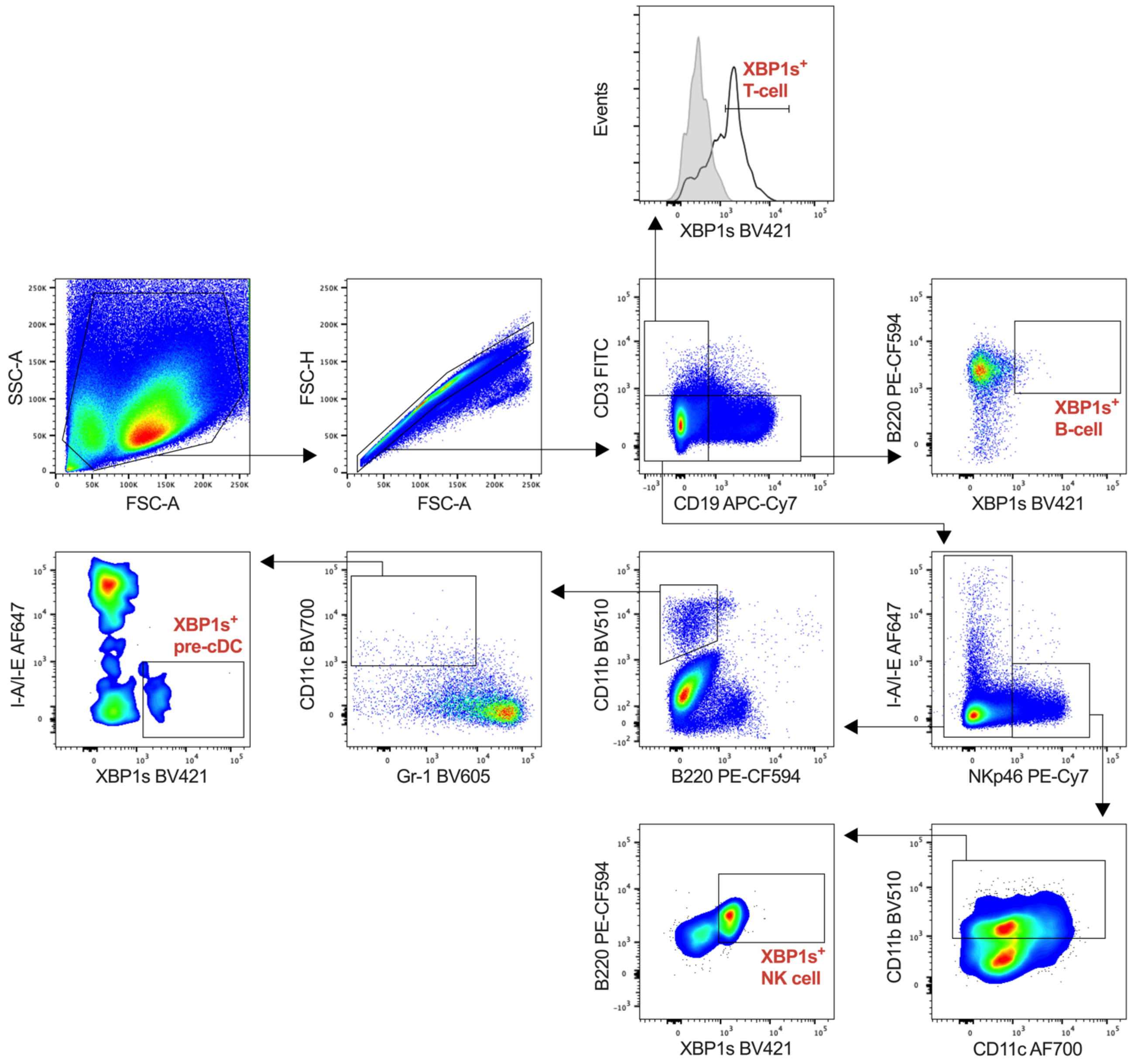

Supplementary Figure 5. Fetal bone marrow XBP1s gating strategy. Gating strategy used to immunophenotypically characterize XBP1s$^+$ populations within fBM. Shaded area of the histogram represents XBP1s BV421 FMO control.

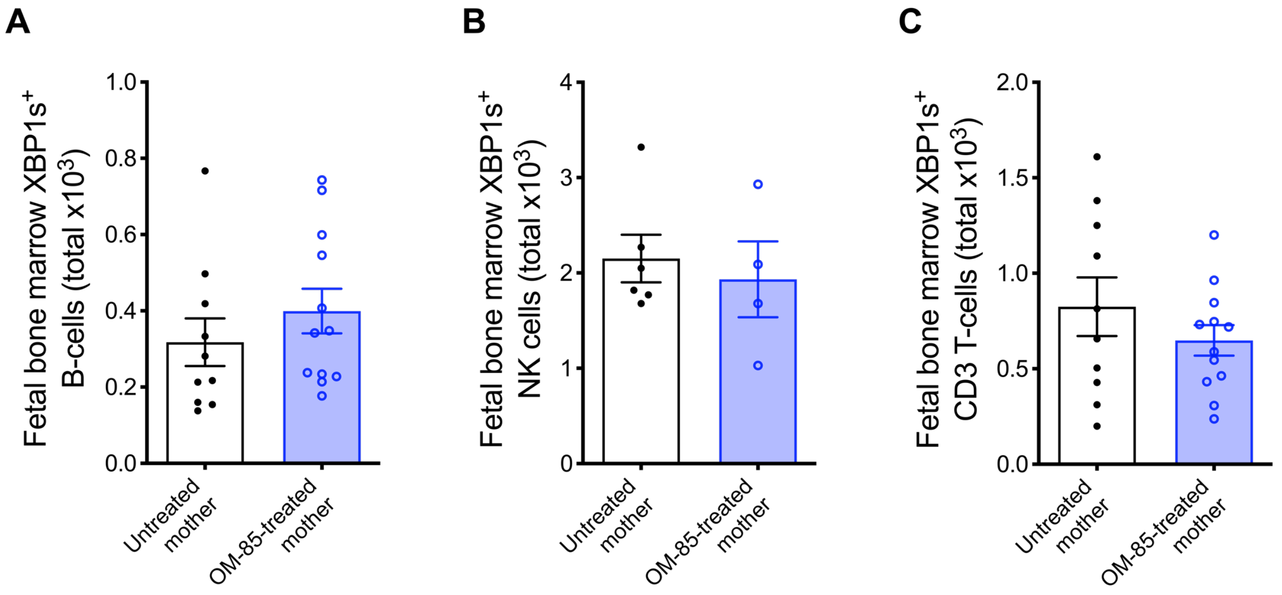

Supplementary Figure 6. XBP1s is expressed in multiple cell types within fetal bone marrow. Absolute numbers of **(A)** $CD19^{+}B220^{+}XBP1s^{+}$ B-cells, **(B)** $NKp46^{+}CD11b^{+}B220^{+}CD11c^{lo}XBP1s^{+}$ NK cells and **(C)** $CD3^{+}XBP1s^{+}$ T-cells in fetal bone marrow. Data are presented from individual animals comparing fetuses from OM-85-treated and untreated mothers and displayed as bar graphs showing mean ± SEM of $n = 4$ independent experiments. Statistical significance was assessed using Mann-Whitney *U* test (A, B) or Student's *t* test (C) based on distribution of the data as determined by D'Agostino-Pearson omnibus normality test.
